# The E-Cadherin Cleavage Associated to Pathogenic Bacteria Infections Can Favor Bacterial Invasion and Transmigration, Dysregulation of the Immune Response and Cancer Induction in Humans

**DOI:** 10.3389/fmicb.2019.02598

**Published:** 2019-11-08

**Authors:** Christian A. Devaux, Soraya Mezouar, Jean-Louis Mege

**Affiliations:** ^1^IRD, MEPHI, APHM, Aix-Marseille University, Marseille, France; ^2^CNRS, Institute of Biological Science (INSB), Marseille, France; ^3^Institut Hospitalo-Universitaire (IHU)-Mediterranee Infection, Marseille, France; ^4^APHM, UF Immunology Department, Marseille, France

**Keywords:** bacterial invasion, bacteria-inducing cancer, pathophysiology, E-cadherin, sheddases

## Abstract

Once bound to the epithelium, pathogenic bacteria have to cross epithelial barriers to invade their human host. In order to achieve this goal, they have to destroy the adherens junctions insured by cell adhesion molecules (CAM), such as E-cadherin (E-cad). The invasive bacteria use more or less sophisticated mechanisms aimed to deregulate CAM genes expression or to modulate the cell-surface expression of CAM proteins, which are otherwise rigorously regulated by a molecular crosstalk essential for homeostasis. Apart from the repression of CAM genes, a drastic decrease in adhesion molecules on human epithelial cells can be obtained by induction of eukaryotic endoproteases named sheddases or through synthesis of their own (prokaryotic) sheddases. Cleavage of CAM by sheddases results in the release of soluble forms of CAM. The overexpression of soluble CAM in body fluids can trigger inflammation and pro-carcinogenic programming leading to tumor induction and metastasis. In addition, the reduction of the surface expression of E-cad on epithelia could be accompanied by an alteration of the anti-bacterial and anti-tumoral immune responses. This immune response dysfunction is likely to occur through the deregulation of immune cells homing, which is controlled at the level of E-cad interaction by surface molecules α_E_ integrin (CD103) and lectin receptor KLRG1. In this review, we highlight the central role of CAM cell-surface expression during pathogenic microbial invasion, with a particular focus on bacterial-induced cleavage of E-cad. We revisit herein the rapidly growing body of evidence indicating that high levels of soluble E-cad (sE-cad) in patients’ sera could serve as biomarker of bacterial-induced diseases.

## Epithelial Cadherin in Cell-to-Cell Adhesion and Cell Activation

Cadherins (cad) belong to the superfamily of cell-adhesion molecules (CAMs) (Takeichi, 1977; Nollet et al., 2000; Angst et al., 2001; Hulpiau and van Roy, 2009). Characterized by their adhesion properties mediated through repeated extracellular cad domains (ECs) under Ca^2+^ control, cad play a key role in cell-to-cell interactions (Hyafil et al., 1981). Several subtypes of cad are encoded in the human genome (Oda and Takeichi, 2011). These molecules were classified according to their tissue distribution: for example, the P prefix is used in P-cad (encoded by *CDH3*) to define placental cad, N-cad (encoded by *CDH2* and *CDH12*) for neural cad, VE-cad (encoded by *CDH5*) for vascular endothelial cad, and E-cadherin (E-cad) (encoded by *CDH1*) for epithelial cad. The *CDH1* gene, located on chromosome 16q22.1, comprises 16 exons and 15 introns (Berx et al., 1995), and it is transcribed into a 4.5Kb pre-mRNA that is spliced to generate the E-cad mRNA. Transcriptional repression of *CDH1* gene is achieved by a range of transcriptional repressors that bind its promoter, including members of the SNAIL and ZEB gene families of zinc-finger transcription factors (Cano et al., 2000; Bolós et al., 2003; Cadigan and Waterman, 2012). Repression of *CDH1* gene can also be the result of CpG-island hypermethylation of its promoter, loss of heterozygosis at 16q22.1, and inactivating mutations (Berx et al., 1998; Lombaerts et al., 2006).

**Figure 1:**
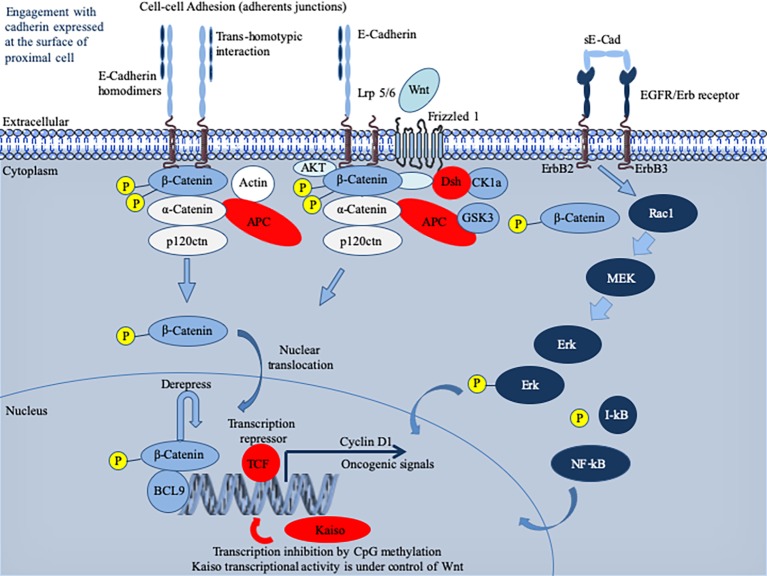
Schematic representation of the E-cadherin (E-cad) interactions and signaling pathway. Newly synthesized E-cad are transported from the Golgi apparatus to the cell surface where they are available to engagement in intercellular interactions. The model presented reflects evidence that E-cad homodimers are involved in adherens’ junctions. Loss of E-cad expression in epithelia results in loosening of intercellular contacts. E-cad regulates the intracytoplasmic pool of α-cat and β-cat acts as a signal transducer molecule in response to upstream Wnt pathway (Fagotto, 2013). Briefly, the Wnt pathway is initiated by the binding of an extracellular Wnt ligand to a surface receptor composed of Frizzled, a seven transmembrane (7TM) molecule and low-density lipoprotein receptor-related protein 5 or 6 (Lrp5/6). As a result of the Wnt pathway activation that mobilizes several intracytoplasmic molecules (including disheveled, adenomatous polyposis coli – APC binds axin and β-cat and inhibits glycogen synthase kinase 3β – and ΑΚΤ kinase) (Fang et al., 2007; MacDonald et al., 2009; Wu et al., 2009), free cytoplasmic β-cat destruction is inhibited and β-cat translocates to the nucleus. Once in the nucleus, β-cat activates expression of genes such as cyclin D1 or c-MYC, otherwise repressed by the T cell factor/lymphoid enhancer factor (TCF/LEF) (Gumbiner, 1995). To achieve trans-activation, β-cat recruits a range of nuclear co-factors including CBP/p300, Brg-1, and the adaptor protein BCL9 (Hecht et al., 2000; Barker et al., 2001; Kramps et al., 2002; de la Roche et al., 2008). Cad-free p120^ctn^ can also trigger the nuclear translocation of β-cat through its association with the Vav2 small GTPase and activation of JNK kinase (Wu et al., 2009; Valls et al., 2012). It is worth noting that TCF/LEF-binding sites have been identified in the *CDH1* gene promoter, which may permit a feedback control loop where β-cat might activate E-cad mRNA production (Huber et al., 1996). E-cad expression can also be regulated by microRNAs (not shown), such as miR200, which favor E-cad mRNA expression (Gregory et al., 2008). Cells with greater E-cad abundance can sequester and thereby inhibit the ability of β-catenin to translocate to the cell nucleus to derepress the activity of its DNA-binding factor TCF/LEF. When unable to engage in interactions, E-cad enters an endocytic uptake and is directed to early sorting endosomes from which they can either be recycled back to the cell surface by recycling endosomes or routed from late endosomes to proteasome where they undergo degradation (for details see the reviews by (Niessen et al., 2011; Nava et al., 2013; McCrea et al., 2015). During infection with bacteria, the pathogen can either regulate the expression of the *CDH1* gene and thereby the cell-surface abundance of E-cad or trigger the catalytic cleavage of E-cad. This can result in the release of a cytosolic pool of β-catenin acting as a downstream effector in the Wnt signaling pathway and induction of oncogenic signals. The sE-cad can associate with intact E-cad present on other cells to alter Cad-dependent cellular behavior. It can also interact with the EGFR molecule to activate the ERK signaling pathway.

Initially described as liver cell adhesion molecule (L-CAM) and uvomorulin (Gallin et al., 1983; Schuh et al., 1986), E-cad is a single-pass type I transmembrane glycoprotein of 120 kDa that plays a major role in cell polarity, intercellular adhesion, and tissue integrity (Ogou et al., 1983; Niessen et al., 2011; van Roy, 2014). It possesses five EC repeats with binding sites for Ca^2+^ (Shapiro et al., 1995). These predominantly homophilic E-cad dimerize in cis at the cell’s surface and the homodimer can then interact in trans with an adjacent E-cad homodimer on a neighboring epithelial cell to form adherens junctions (Boggon, 2002). However, E-cad can also exhibit heterophilic interactions in trans with the α_E_β_7_ integrin, also called CD103 antigen of T-lymphocytes, which generally lacks E-cad cell surface expression (Cepek et al., 1994; Sheridan and Lefrançois, 2011) as well as it can bind the killer cell lectin receptor G1 (KLRG1) expressed on T-lymphocytes and natural killer (NK) cells (Kilshaw, 1999; Ito et al., 2006). Over-expression of E-cad can delay the rate of cell migration (Hermiston et al., 1996). Loss of E-cad can reduce CD103^+^ T-cell antitumor activity (Shields et al., 2019). Under physiological conditions, E-cad interacts with p120-ctn and β-catenin (β-cat) *via* its intracytoplasmic tail (Nagafuchi and Takeichi, 1988; McCrea and Gumbiner, 1991; Kourtidis et al., 2013). The cytoplasmic tail of E-cad consists of the juxta membrane domain (JMD), which allows the clustering of cad and contributes to the adhesive strength *via* p120-ctn, and the cat-binding domain (CBD), which interacts with β-cat and γ-cat (Kemler, 1993; Yap et al., 1998). The α-cat links the bound β-cat and the actin cytoskeleton. Signaling through E-cad cytoplasmic tail is a complex process which involves multiple contacts with intracytoplasmic partners, whose diversity is just beginning to be elucidated by the characterization of the E-cad interactome (Guo et al., 2014). E-cad is a tumor suppressor acting through intracytoplasmic retention of β-catenin stocks and suppresses inflammatory signaling pathways (Figure 1).

## E-Cadherin and Other Cadherin/Cell Adhesion Molecules Used as Target Receptors for Bacteria

*Fusobacterium nucleatum*, a pathogen associated with oral plaque formation and colorectal cancers, binds E-cad through its FadA adhesin (Rubinstein et al., 2013). This interaction up-regulates the Annexin A1 and activates β-cat signaling (Zhou et al., 2018; Rubinstein et al., 2019). The *F. nucleatum* was reported to be associated with a specific epigenetic pattern of tumor cells characterized by hypermethylation of CpG islands, high MSI and MLH1 hypermethylation (epigenetic silencing), and up-regulation of microRNA-21 (Tahara et al., 2014; Yang et al., 2017). *Listeria monocytogenes,* the causative agent of severe food poisoning, which sometimes lead to meningitis, internalizes when internalin A (InlA) and InlB bind to E-cad and the hepatocyte growth factor receptor on the basolateral surface of epithelial cells (Cossart and Sansonetti, 2004; Barbuddhe and Chakraborty, 2009; Ortega et al., 2017). *Streptococcus pneumoniae* can cause pneumonia, meningitis, and bacteremia. The flamingo cadherin was reported to serve as receptor for the *S. pneumoniae* fructose bisphosphate aldolase (Blau et al., 2007), and E-cad was found to act as adherence receptor for the pneumococcal surface adhesin A (PsaA) of *S. pneumoniae* during the colonization of nasopharyngeal epithelial cells (Anderton et al., 2007). *Helicobacter pylori*, a bacterium responsible for severe gastric disease, adhere to target cells through interaction with CEACAM cell-surface receptor *via* its HopQ adhesin (Javaheri et al., 2016). Then, the bacterial HtrA sheddase cleaves the gastric epithelial cell-to-cell junctions through endoproteolysis of E-cad, occludin, and claudin-8 (Tegtmeyer et al., 2017). After transmigration of *H. pylori* to the basolateral membrane of gastric epithelial cells, the T4SS pilus is activated and injects the CagA cytotoxin into the target cell where the release of β-cat is stimulated (Suzuki et al., 2005; Murata-Kamiya et al., 2007) (Zhao et al., 2018; Tegtmeyer et al., 2019).

In the world of bacteria, other cadherin and CAM play a role during the phase of attachment and invasion (Table 1). The attachment of *Leptospira interrogans* to host cells was found to be mediated through its interaction with VE-cad, which triggers the process leading to different symptoms, including liver dysfunction, kidney failure, myocarditis, and sometimes the pulmonary hemorrhagic manifestations of leptospirosis (Evangelista et al., 2014). Human host colonization by *Haemophilus influenzae* began with the binding of the bacteria to I-CAM1 on the surface of the respiratory tract epithelial cells through its Type IV pilus (Tfp), a process leading to respiratory diseases such as cystic fibrosis or chronic obstructive pulmonary disease (Novotny and Bakaletz, 2016). *H. influenzae* invasion is made even more effective as humans carry adenovirus or respiratory syncytial virus, which are known to increase cell-surface expression of I-CAM1. *H. influenzae* can also bind CEACAM through outer membrane protein OMP-1 (Tchoupa et al., 2015). CEACAM was also reported to serve as a receptor for the Opa protein of *Neisseria gonorrhoeae* during the colonization of urogenital mucosal surfaces in humans. It can progress toward acute urethritis with purulent urethral discharge in men, while the infection can remain asymptomatic in women or evolve toward an inflammation of the endocervix or an infection of fallopian tubes (Sintsova et al., 2015).

**Table 1:** Function of E-cad and other CAM molecules in bacteria-mediated infectious diseases.

Bacteria	Receptor(s)	Interaction receptor/pathogen	References
*Listeria monocytogenes*	E-cadherin	Entry receptor	Bonazzi et al. (2009)
*Helicobacter pylori*	CEA-CAM (E-cadherin)	Induce E-cad cleavage, β-cat signaling	Murata-Kamiya et al. (2007); Hoy et al. (2010); Tegtmeyer et al. (2019)
*Fusobacterium nucleatum*	E-cadherin	Bacteria receptor, β-cat signaling	Rubinstein et al. (2013, 2019); Ma et al. (2018)
*Bacteroides fragilis*	E-cadherin	Bacteria receptor induce E-cad cleavage	Chambers et al. (1997); Obiso et al. (1997)
*Campylobacter jejuni*	E-cadherin	Induce E-cad cleavage, transmigration	Boehm et al. (2015)
*Streptococcus pneumoniae*	E-cadherin, flamingo-CAM	Bacteria adhesion	Anderton et al. (2007)
*Leptospira interrogans*	VE-cadherin	Bacteria adhesion	Evangelista et al. (2014)
*Haemophilus influenza*	ICAM-1, CEA-CAM	Bacteria adhesion	Bookwalter et al. (2008)
*Haemophilus influenza*	ICAM-1, CEA-CAM	Bacteria adhesion	Bookwalter et al. (2008)
*Neisseria meningitidis*	CEA-CAM	Entry receptor	Griffiths et al. (2007)
*Yersinia pseudotuberculosis*	β1-integrin	Bacteria adhesion and internalization	Isberg and Leong (1990)

*CAM, cell adhesion molecule; V-CAM, vascular cell adhesion molecule; ICAM, intercellular adhesion molecule; NCAM, neuronal cell adhesion molecule; CEA-CAM, carcinoembryonic antigen-related cell adhesion molecule*.

Besides bacteria, many human pathogens also use E-cad and/or other CAM during the human host colonization, indicating the ubiquitous nature of this process. For example, a fungus, *Aspergillus fumigatus*, which is responsible for the majority of invasive mold infections in patients undergoing chemotherapy or organ transplantation, was found to bind E-cad and to use it as a receptor for adhesion and endocytosis of blastopores in epithelial cells (Xu et al., 2012; Yan et al., 2015), and *Candida albicans*, the causative agent of hematogenously disseminated and oropharyngeal candidiasis, internalizes through direct interactions between its surface adhesin Als3 and E-cad on the target cell (Egusa et al., 2005; Phan et al., 2007). Regarding viruses, it has been reported that E-cad, together with claudin 1 and occludin, plays a role in the hepatitis C virus entry into hepatocytes (Colpitts et al., 2016; Li et al., 2016). Several CAM, such as I-CAM, V-CAM, and N-CAM have also been identified as viral receptors for viruses, such as coxsackie A virus; rhinovirus, Enterovirus D68, encephalomyocarditis virus, and rabies virus, respectively (Thoulouze et al., 1998; Bhella, 2015; Wei et al., 2016).

## E-Cadherin Degradation Induced by Bacteria During Tissues Invasion and Transmigration

Usually, the commensal microbiota, which supplies the host with molecules essential to life and shapes gene expression in eukaryotic cells (Devaux and Raoult, 2018), together with epithelial cell barriers and appropriate immune responses, efficiently protects the internal body against pathogenic microbial invasion (Abt and Pamer, 2014). Pathogenic bacteria have engineered different strategies to get around these natural defenses by the transcellular route, by acting on cell-to-cell junctions, or by taking advantage of damaged tissues.

**Figure 2:**
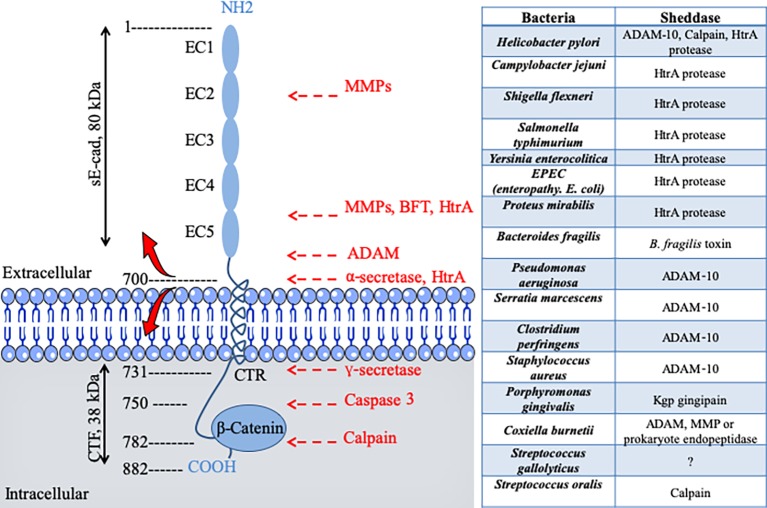
Schematic diagram of E-cad and its cleavage sites by proteases after pathogenic bacteria infection. E-cad is a transmembrane protein containing five extracellular repeated domains (EC1 to EC5), a transmembrane region, and an intracytoplasmic C-terminal region (CTR). The extracellular portion of E-cad forms junction with CAM on proximal cell (see Figure 1), whereas the CTR binds β-catenin and other signaling molecules. Left panel, catalytic enzymes have the capacity to cleave E-cad at specific sites indicated by red arrows. Cleavages occur either in the intracellular CTR of the molecule (e.g., caspase 3 or calpain) generating polypeptides that are capable of triggering signals, or in the extracellular portion of the molecule (e.g., the eukaryotic sheddases MMP and ADAM or the prokaryotic sheddases: BTF or HtrA), leading to soluble extracellular E-cad fragment release. For instance, the eukaryotic ADAM sheddases catalyze a cleavage of E-cad that results in the release of the 80-kDa soluble ectodomain form sE-cad and a 38-kDa C-terminal fragment (CTF). The prokaryotic HtrA serine protease can also cleave E-cad at different extracellular sites. Right panel bacteria reported to trigger E-cad cleavage (and release of sE-cad) and identification of sheddases involved in this process.

*Clostridium perfringens*, which is the causative agent of gas gangrene and food poisoning, produces a pore-forming delta toxin, which was found capable of reducing cell surface expression of E-cad by enhancing ADAM-10 sheddase activity (Figure 2; Seike et al., 2018). Similarly, the alpha toxin of *Staphylococcus aureus* binds to and up-regulates ADAM-10 metalloprotease activity in alveolar epithelial cells. This activity results in the cleavage of E-cad and contributes to the pathogenesis of lethal pneumonia (Inoshima et al., 2011). *Clostridium botulinum* produces the botulinum neurotoxin (BoNT), which provokes flaccid paralysis known as botulism, by inhibiting neurotransmitter release at the neuro-muscular junctions (Carter and Peck, 2015). To allow BoNT complex to pass through the epithelial barrier of the intestinal tract and act on the neurotransmission process, one compound of the BoNT complex termed hemagglutinin (HA), binds E-cad and disrupts the tight junctions (Sugawara and Fujinaga, 2011). The prokaryotic high temperature requirement A (HtrA) protease-mediated cleavage of E-cad that precedes the process of transmigration has been described for gastrointestinal pathogens, such as enteropathogenic *Escherichia coli*, *Shigella flexneri*, and *Campylobacter jejuni* (Boehm et al., 2012; Hoy et al., 2012; Elmi et al., 2016). The HtrA sheddase of *Helicobacter pylori* was found to open adherens junctions by cleaving E-cad and claudin-8 occludin (Tegtmeyer et al., 2017). HtrA sheddase has been found in most of the bacterial genomes studied to date and is associated with pathogenicity. An opportunistic pathogen like *Serratia marcescens* produces a pore-forming toxin (ShlA) responsible for the tissues damage required to cross cellular barriers (Hertle and Schwarz, 2004). Another opportunistic bacterium, *Pseudomonas aeruginosa*, which causes aggressive infections in patients compromised by respiratory diseases such as cystic fibrosis, also encodes a pore-forming toxin (exolysin A) that induces major injuries of tissues (Reboud et al., 2017). Both SHlA and ExlA influence ADAM-10 activation triggering E-cad and VE-cad cleavage in epithelial and endothelial cells, respectively, as well as soluble CAMs shedding, and intercellular junction rupture (Reboud et al., 2017). *Leptospira interrogans*, which crosses host tissue barriers and causes leptospirosis, secretes a protein named LIC10831 that binds E-cad and VE-cad and plays a role during bacterial invasion (Eshghi et al., 2019). The genome of *Porphyromonas gingivalis*, a bacterium associated with adult periodontitis (Katz et al., 2000), encodes three cysteine proteases named Gingipains (HRgpA, RgpB, and Kgp). The Kgp protease was found capable to disrupt adherens junction by cleavage of E-cad (Katz, 2002). *P. gingivalis* also cleaves N- and VE-cads (Sheets et al., 2005). With the *L. monocytogenes*, the infectious process starts with the interaction between the invasion proteins internalin and InlB and their cellular receptor E-cad and hepatocyte growth factor receptor (HGF-R)/Met (Seveau et al., 2004). E-cad also constitutes a target for *L. monocytogenes* in order to disrupt the blood brain barrier and facilitate the invasion of the brain (Al-Obaidi and Desa, 2018). In *Chlamydia trachomatis* infections, a bacterium responsible for acute salpingitis and cervicitis, which can also induce scarring disease of the ocular mucosa, a DNA methylation of the *CDH1* promoter and downregulation of E-cad expression, was reported (Rajić et al., 2017). It can be hypothesized that the list of pathogenic bacteria shown to cleave the E-cad during the invasion process will increase rapidly.

For other pathogens, such a requirement to achieve transmigration for colonizing their host can also be illustrated with a few examples. Tissues invasion by *C. albicans* is associated with degradation of E-cad mediated by the fungus aspartyl proteinase Sap5p under the control of the transcription factor Rim101p (Villar et al., 2007). *C. albicans* was also reported capable of reducing E-cad mRNA expression (Rouabhia et al., 2012). More recently, it was reported that *C. albicans* synergized with *Streptococcus oralis* to increase the proteolytic degradation of E-cad by μ-calpain, which facilitates fungal invasion (Xu et al., 2016). It illustrates the fact that the modulation of cell-surface expression of E-cad is not limited to bacteria, but is rather a general mechanism used by infectious pathogens for invasion and transmigration (Grabowska and Day, 2012).

## Proteolytic Cleavage of E-Cadherin by Eukaryotic Sheddases

As described above in this paper, pathogenic bacteria such as *H. pylori*, *Pseudomonas aeruginosa*, *Serratia marcescens*, *Clostridium perfringens*, and *S. aureus* have developed takeover stratagems to use eukaryotic sheddases of the human host (e.g., ADAM-10) in order to modulate the host cell surface expression of E-cad.

**Figure 3:**
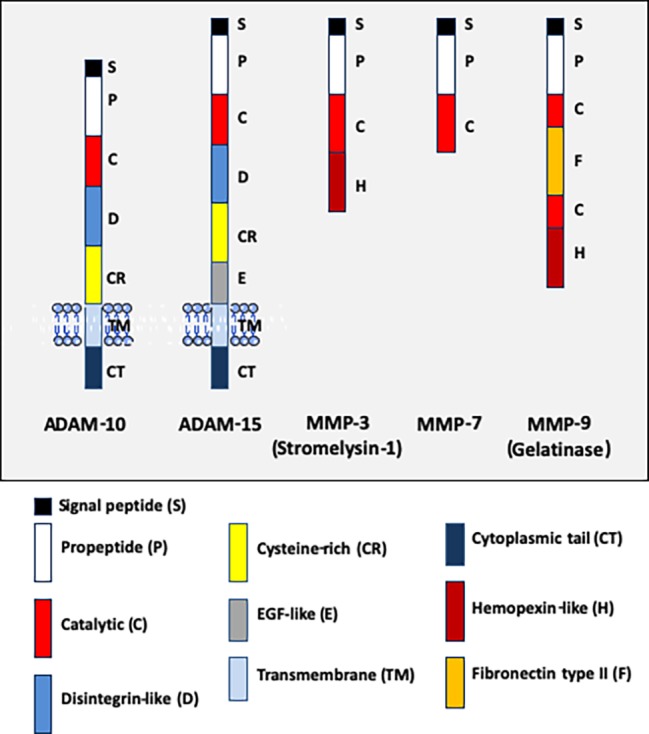
Schematic representation of the structural organization of some human ADAM and MMP pro-enzyme sheddases. The ADAM molecules (ADAM-10 and ADAM-15) are transmembrane proteins, whereas the MMP (MMP-3, MMP-7, and MMP-9) are soluble proteins. It should be noted (not shown) that several MMP are anchored at the cell-surface by a transmembrane domain (e.g., MMP-14 or MMP-15), by a glycosylphosphatidylinositol anchor (e.g., MMP-17 or MMP-25) or by an amino-terminal signal anchor (e.g., MMP-23). From the NH_2_-terminal extremity to the COOH-terminal extremity, ADAM-10 (748 a.a. residues) and ADAM-15 (772 a.a. to 863 a.a. residues depending the isoform) contain a signal peptide, a pro-peptide, a metalloprotease domain with catalytic activity, a disintegrin-like domain, a cysteine-rich domain, a transmembrane domain, and a cytoplasmic tail. ADAM-15 is characterized by the presence of an EGF-like domain taking place between the cysteine-rich domain and the transmembrane polypeptide. MMP-3 (or stromelysin-1, a 477 a.a. residues pro-enzyme protein of 51-kDa which can be activated in the 43-kDa mature catalytic form by removal of the pro-domain) has a basic MMP structure with a signal peptide, a pro-peptide, a catalytic domain, a hinge region, and an hemopexin domain. MMP9 (or gelatinase B, type IV collagenase, a 707 a.a. residues pro-enzyme protein of 92-kDa which can be activated as a 83-kDa mature enzyme following removal of the pro-domain), contains a signal peptide, a pro-peptide, a catalytic domain, a fibronectin type II domain, a second catalytic domain, an hinge region, and an hemopexin domain.

The cleavage of adhesion molecules is far from being limited to the pathogens' invasion processes. Apart dysbiosis, proteolysis is a common physiological mechanism of post-translational regulation that affects 2–4% of the proteins expressed on the surface of cells (Pandiella et al., 1992; Arribas and Merlos-Suárez, 2003). E-cad is one of the molecules that can undergo proteolytic cleavage (both intracellular and extracellular), providing an alternative regulatory mechanism to reduce its cell surface expression (Noë et al., 2001; Marambaud, 2002; van Roy and Berx, 2008). It is essential to regulate the balance between adhesion and migration of cells. The human genome encodes almost 600 proteases, which control a wide range of processes essential to life (Turk et al., 2012). Proteases can be organized into five main classes, including cysteine proteases, serine proteases, metalloproteases, threonine proteases, and aspartic proteases, with approximately one half being extracellular and the other half intracellular. Quantitative cell surface expression of E-cad is therefore determined by the balance between biosynthesis, trafficking, transfer to cell-surface, intracellular and extracellular proteolytic cleavage, and intracellular degradation, and these processes are considered crucial determinants for cell behavior (Godt and Tepass, 1998). Endoproteases (which cleave internal peptides bonds), which are capable of extracellular E-cad cleavage, belong to the large family of sheddases (Grabowska and Day, 2012). The human sheddases (Figure 3) include zinc-dependent matrix metalloproteases (matrilysin/MMP-2, 3, 7, 9, and 14) (Lee et al., 2007; Symowicz et al., 2007; Klein and Bischoff, 2011), members of the disintegrin metalloproteases family (adamalysin/ADAM-10 and -15) (Maretzky et al., 2008; Najy et al., 2008; Giebeler and Zigrino, 2016), cysteine cathepsins (B, L, S) (Jordans et al., 2009), serine protease kallikrein (KLK-6 and -7) (Johnson et al., 2007; Klucky et al., 2007), plasmin serine protease (Ryniers et al., 2002), and the membrane-bound aspartic proteinases BACE-1 and BACE-2 (Wakabayashi and De Strooper, 2008; Grabowska and Day, 2012). Sheddases are secreted as proenzymes and become mature after processing of the propeptide; for example, the matrix metalloprotease 3 is synthesized as a zymogen (proMMP-3) which is converted to full activity (two active forms of 45-kDa and 25-kDa) by limited proteolysis mediated by elastase and cathepsin G (Okada and Nakanishi, 1989; Becker et al., 1995). Similarly, the MMP-9 is synthesized as a 92-kDa proMMP-9, and then the active MMP-3 initially cleaves proMMP-9 to generate a 86-kDa intermediate ultimately cleaved to convert the intermediate form into a 82-kDa catalytic form (Ogata et al., 1992). The catalytic domains of MMP-9 alone can hydrolyze non-collagenous proteins and synthetic substrates, but cannot cleave triple helix collagens without the hemopexin domain (Nagase et al., 2006). Its fibronectin type II domains are important to cleave type IV collagen, elastin, and gelatins. The hemopexin domain is required for collagenolytic activity of the collagenase. The catalytic activity of sheddases triggers the extracellular release of a soluble E-cad (sE-cad) fragment of about 80-kDa from the cell surface. This process is accompanied by the simultaneous delivery of free β-cat into the cell cytosol, which then translocates into the cell nucleus where it contributes to the modulation of gene expression. It is worth noting that sE-cad might also behave as a signaling molecule through ErbB receptor activation (Najy et al., 2008). As already mentioned above, some pathogenic bacteria can enslave eukaryotic sheddase to get E-cad cleaved.

## Inactivation of the *CDH1* Gene by Methylation is Associated With Pre-carcinogenic Programming During Pathogenic Bacteria Invasion

Several pathogenic bacteria were found to control the *CDH1* gene expression at the chromatin level through activation of signaling cascade leading to modulation of DNA-binding proteins or directly in the nucleus through epigenetic modifications (Bierne et al., 2012). Inactivation of the *CDH1* gene leads to the down-modulates of E-cad protein expression.

It was reported that methylation of the *CDH1* gene promoter is a frequent event in samples from *H. pylori* infected patients with chronic gastritis, suggesting that *CDH1* inactivation is an early step toward gastric tumorigenesis (Kague et al., 2010). Methylation of the *CDH1* gene promoter was also reported in about 30–40% of patients with *H. pylori*-associated gastric carcinoma (Bahnassy et al., 2018). The *CDH1* promoter methylation was reduced after *H. pylori* eradication (Perri et al., 2007). *Chlamydia trachomatis* infection inducing scarring disease of the ocular mucosa was found to be associated with *CDH1* promoter DNA methylation and down-regulation of E-cad (Rajić et al., 2017). *Acinetobacter baumannii* is an opportunistic pathogen causing severe diseases in patients with mechanical ventilation. The nuclear targeting of *Acinetobacter baumannii* transposase (Tnp) induces DNA methylation of CpG regions in the promoter of the *CDH1* gene resulting in down-regulation of gene expression (Moon et al., 2012). To date, the *CDH1* gene inactivation has not been systematically explored for pathogenic bacteria and should be questioned as part of the exploration of the molecular mechanisms by which pathogenic bacteria deregulate E-cad expression to colonize the human host.

The epigenetic modulation of the *CDH1* gene during pathogenic bacteria infection mimics processes well-described in the studies regarding the embryologic development. Repression of the *CDH1* gene and cad-switching from E-cad to N-cad were considered essential for transitioning away from pluripotency (Sauka-Spengler and Bronner-Fraser, 2008; Li et al., 2010; Pieters and van Roy, 2014). Such cad-switching (Thiery, 2002) as well as reduced expression of E-cad have also been observed in cancer cases (including breast cancer, gastric cancer, colorectal cancer, and hepatocellular carcinomas), indicating that inhibition of E-cad surface expression can play a central role in the progression toward cancer (Jeanes et al., 2008; Gall and Frampton, 2013). However, in lung cancer cells, E-cad was found induced by WNT7a (Ohira et al., 2003), and E-cad expression is increased in both ovarian cancer malignant effusions and solid metastases (Davidson et al., 2000; Elloul et al., 2006). E-cad is generally considered a tumor suppressor through inhibition of β-cat nuclear translocation (Gottardi et al., 2001) and can act as oncogene through binding to EGFR/Erb receptor that triggers ERK and AKT signaling pathways providing advantage for cancer development and metastasis (Wells et al., 2008; Rodriguez et al., 2012).

With regard to viruses as examples of other pathogens modulating *CDH1* gene expression, it has been reported that hepatitis B virus (HBV) represses E-cad at the transcriptional level by hypermethylation of the *CDH1* promoter on CpG Island 1 with possible consequences on hepatocellular carcinogenesis by promoting detachment of surrounding cells and their migration to the primary tumor site (Lee et al., 2005). Alternatively, loss of E-cad in HBV infected cells can also be regulated at a post-translational level by proteases and SUMOylation (Ha et al., 2016). Hypermethylation that triggers *CDH-1* gene repression was also found with the human papillomavirus (HPV) that increases cellular methyltransferase 1 (Dnmt1) activity *via* its viral E7 protein (Laurson et al., 2010).

## Aberrant Splicing of the E-Cadherin Transcript

Among the gene silencing molecular mechanisms underlying E-cad loss, one involves the expression of nonfunctional truncated *CDH1* gene transcript. E-cadherin mRNA with premature termination codon mutation was reported in chronic lymphocytic leukemia cells (Sharma and Lichtenstein, 2009). This transcript (which lacks the exon 11) plays a role in silencing the production of E-cad. The amounts of wild-type E-cad mRNA inversely correlated with the amounts of aberrant transcript resulting in the up-regulation of the Wnt-β catenin pathway. The production of E-cad mRNA variant by alternative splicing has also been linked to a decrease in cell-cell adhesion and an increase in cell migration (Matos et al., 2017). The novel E-cadherin variant, a truncated soluble form of E-cad resulting from a deletion of the first 34 nt in the exon 14 of the *CDH1* mRNA, induces changes characteristic of the Epithelial to Mesenchymal Transition (EMT) process, a key event in tumor progression (Matos et al., 2017). Moreover, the over-expression of this novel E-cad truncated form in transfected cells resulted in downregulation of wild-type E-cad expression. This truncated *CDH1* gene transcript was recently found in breast cancer cells (Rosso et al., 2019). Aberrant splicing of *CDH1* gene transcript (exon 8 or exon 11 skipped aberrant transcripts) was also reported in gastric carcinoma (Garziera et al., 2013; Li et al., 2015). Although this is a possibility, it has not yet been demonstrated that the expression of a *CDH1* aberrant transcript in gastric cancers is controlled by *H. pylori*. However, it is known that aberrant splicing of cellular gene transcripts can occur during bacterial invasion (Sun, 2017). Indeed, massive alterations in the pattern of cellular mRNA splicing were reported upon infection with bacteria such as *Anaplasma phagocytophilum*, the etiologic agent of the human granulocytic anaplasmosis (Dumler et al., 2018) and *Mycobacterium tuberculosis* the etiologic agent of tuberculosis (Kalam et al., 2018). This may be consistent with the observation that *Mycobacterium tuberculosis* induced an epithelial mesenchymal transition in a pulmonary adenocarcinoma epithelial cell line (Gupta et al., 2016), a phenomenon orchestrated at the level of E-cad cell-surface expression. It was also reported that the EMT of mesothelial cells occurred in *Mycobacterium tuberculosis*-associated pleurisy, together with a reduction in E-cad expression (Kim et al., 2011). Moreover, expression level of E-cad was reported to differ between pulmonary tuberculosis patients and latent tuberculosis individuals (Sun et al., 2018).

## Release of Soluble E-Cadherin After Bacterial Infection as an Early Event Toward Carcinogenesis

Aside from the transcriptional repression of the *CDH1* gene, another interesting mechanism, which could interfere with the anti-bacterial defenses of the host, is the release of sE-cad in body fluids following a sheddase-mediated cleavage of E-cad. The combination of sE-cad release together with other pro-inflammatory factors was highlighted as a triggering signal that promotes gastric adenocarcinoma or colorectal tumors in patients infected with *H. pylori*, *B. fragilis*, *or Streptococcus gallolyticus* (Wu et al., 2003; O’Connor et al., 2011; Kumar et al., 2017; Chung et al., 2018).

*H. pylori*, a bacterium that colonizes the gastric mucosa, is known as a risk factor for the development of chronic atrophic gastritis, gastroduodenal ulcers, and adenocarcinoma. An *in vitro* experimental model of cell transfection has demonstrated that *H. pylori* triggers β-cat activation through the interaction of virulence factor CagA with E-cad (Murata-Kamiya et al., 2007). O’Connor et al. (2011) reported that *H. pylori* induced the activation of host protease calpain *via* the toll-like receptor 2 (TLR2) and disruption of gastric epithelium. Another report suggests that ADAM-10 is induced in *H. pylori* infection and contributes to the shedding of E-cad (Schirrmeister et al., 2009). It was also reported that a serine protease HtrA from *H. pylori* mediate direct cleavage of E-cad (Hoy et al., 2010; Schmidt et al., 2016). Bacterium *S. gallolyticus* has a strong association with colorectal cancer with increased levels of β-cat and c-Myc (Kumar et al., 2017). The genome of another bacterium, *Bacteroides fragilis*, encodes a sheddase, the *B. fragilis* toxin (BFT) also termed fragilis (FRA), which cleaves E-cad and is associated with C-Myc expression and cellular proliferation (Wu et al., 1998, 2003; Remacle et al., 2014; Chung et al., 2018). Chung and co-workers uncovered a complex mechanism whereby the *B. fragilis* toxin (BFT)-mediated cleavage of E-cad initiates a multi-step inflammatory cascade requiring β-cat, IL-17R, NF-B, and Stat3 signaling in colonic epithelial cells. IL-17 dependent NF-κB activation in colic epithelial cells induces a mucosal gradient of C-X-C chemokines that initiates pro-tumoral myeloid cell infiltration to the distal colon and colon cancer. All these cancers are solid tumors. However, a role for sE-cad in the initiation of a pro-carcinogenic process might also be considered. Over the past 3 years, we have reported results suggesting that the bacterium *Coxiella burnetii*, responsible of Q fever, was associated with a higher frequency of Non-Hodgkin Lymphoma (NHL) in *C. burnetii*-infected patients compared to the general population (Melenotte et al., 2016). Recently, we investigated the transcriptional signature that could be associated with the development of NHL in Q fever patients and found an over-expression of genes involved in anti-apoptotic process and a repression of pro-apoptotic genes (Melenotte et al., 2019). Since cell surface expression of E-cad and release of sE-cad have been associated to various pathogenic bacteria known for inducing solid tumors, we have also investigated the levels of expression of these molecules in Q fever patients and observed a significant release of sE-cad in their sera and a down-regulation of E-cad mRNA expression. The sE-cad levels were found increased in the sera of acute and persistent Q fever patients, whereas they remained at the baseline in the control groups of healthy donors, people cured of Q fever, patients suffering from unrelated inflammatory diseases, and past Q fever patients who developed NHL (Table 2). Consequently, sE-cad could be considered a new biomarker of *C. burnetii* infection rather than a marker of NHL-associated to Q fever (Mezouar et al., 2019). We do not yet know which eukaryotic or prokaryotic sheddase could be responsible for the cleavage of E-cad in patients infected with *C. burnetii*. Preliminary studies on peripheral blood mononuclear cells exposed to heat-inactivated *C. burnetii* suggested variations in ADAM-10 and MMP-9 expression. Microarray performed on samples from macrophages and dendritic cells (DC) infected *in vitro* by *C. burnetii* revealed an over-expression of MMP-3 in *C. burnetii*-infected DC. Preliminary investigations in search of sheddase have also been conducted. We tried to blast the protein sequences of more than 20 sheddases known to catalyze the cleavage of E-cad against the hypothetical proteins of four strains of *C. burnetii*. We found that three eukaryotic sheddases (MMP-3, MMP-9, and ADAM-15) presented sequence similarities with bacterial proteins. Experiments are under progress to identify whether an ADAM or MMP eukaryotic protease or a prokaryotic protease encoded by *C. burnetii* could be responsible for sE-cad release in Q fever patients.

**Table 2:** Soluble Cad molecules released in body fluids during bacteria-associated infectious diseases.

Pathogens	Sample/Technic	Cadherin	Results	References
*C. burnetii*	Plasma/Elisa	sE-cadherin	Increased in infected patients including acute and persistent forms of the disease	Mezouar et al. (2019)
*H. pylori*	Serum/Elisa	sE-cadherin	Increased in positive *H. pylori* patients	O’Connor et al. (2011)
*E. coli* (Shiga toxin 2 from)	Plasma/Elisa	VE-cadherin	Increased in infected patients	Doulgere et al. (2015)

*If the general trend is to increase the soluble forms of cadherin molecules in body fluids of patients infected with pathogenic bacteria, caution should be exercised when comparing results obtained with different diagnostic kits, in different laboratories and with serum or plasma samples. Regarding the results reported in the papers mentioned in this table, the concentrations of soluble Cad measured in the samples were two times higher in the infected patients than in healthy controls. For example, in the plasma of *C. burnetii*-infected patients, the sE-cad concentration was about 170–450 ng/ml in acute Q fever (median 272 ng/ml), 260–530 ng/ml in persistent Q fever (median 333 ng/ml), while it was 80–320 ng/ml (median 164 ng/ml) in healthy controls*.

Care must be taken not to overinterpret the significance of sE-cad increase in body fluids during bacterial infections. It is possible that sE-cad release in body fluids corresponds to a ubiquitous phenomenon induced during the process of colonization of the host by a pathogenic bacterium. We already know that it is not limited to bacteria since there is an apparent correlation between the levels of sE-cad in human immunodeficiency virus (HIV)-positive patients and their viral titers (Streeck et al., 2011). Moreover, abnormal concentrations of sE-cad in patients’ sera has been observed in several metabolic and inflammatory diseases (Pittard et al., 1996; Jiang et al., 2009; Shirahata et al., 2018; Sato et al., 2019). High concentration of sE-cad has also been found associated with cancer progression (Grabowska and Day, 2012; Salama et al., 2013; Repetto et al., 2014). For example, high concentration of sE-cad was described in prostate cancer patients and was associated with an over-expression of MMP-2 and MMP-9 (Kuefer et al., 2005; Biswas et al., 2010; Tsaur et al., 2015). Similarly, increased expression of sE-cad was reported in gastric cancer that was associated with the over-expression of MMP-7 (Lee et al., 2006, 2007). Serum levels of sE-cad were increased in patients with ovarian carcinoma the cleavage of E-cad being mediated by MMP-9 (Gadducci et al., 1999; Symowicz et al., 2007). Altogether, these data suggest that high sE-cad concentration in body fluids could simply behave as a factor that predisposes to inflammation and development of cancers, as described for *H. pylori*-infected patients.

## Dysfunction of the Immune Response Orchestrated by Bacteria-Induced E-Cadherin Cleavage

The immune system naturally provides an anti-infectious surveillance at the epithelium level *via* cells that express cell-surface molecules, such as CD103 and KLRG1, able to bind the E-cad found at the surface of epithelial cells. These immune cells are not heavily engaged, unless the other defenses have failed. For example, under normal conditions, the intestinal epithelium is protected by a mucus layer that acts as host defense against microbial attachment (Kim and Ho, 2010; Desai et al., 2016). Upon infection, the specialized intestinal Paneth cells secretes antimicrobial proteins and the commensal intestinal microbiota competes with the infectious pathogens, thereby acting as first line of innate defense to fight against pathogenic bacteria (Cash et al., 2006; Mason and Huffnagle, 2009; Vazeille et al., 2011; Bel et al., 2017). The epithelium integrity controlled by the homotypic interaction of E-cad in trans represents a second barrier protecting the host against intruder transmigration (Kim et al., 2010). When pathogenic bacteria had evaded epithelial cell autophagic clearance and dead cells renewal (Yoshikawa et al., 2009; Benjamin et al., 2013), the host’s immune system represents the last rampart before the pathogens can breach the epithelium and disseminate deeper.

The intestinal villous microfold cells (M cells) are specialized epithelial cells of the gut-associated lymphoid tissues (GALT) that deliver luminal antigens to the underlying immune system after their transport to the basolateral membrane of M cells (Miller et al., 2007). An efficient immune response against a microbial attack requires the migration of cells of the host immune system in the microenvironment where infection occurs and the sequential detection of stress signals, tissue damages and conserved bacterial molecules termed pathogen-associated molecular patterns (PAMPs) (Broz and Monack, 2013). PAMPS include molecules as diverse as lipopolysaccharide, flagellin, peptidoglycan, lipoproteins, and unique bacterial nucleic acid structures. Upon detection of bacterial invasion, host cell receptors such as toll-like receptors and C-type lectin receptors, activate signaling pathways that govern the production of inflammatory cytokines including the IL-1β and IL-18 that can restrict bacterial replication. Causative agents of infectious diseases are therefore characterized by their capacity to elaborate mechanisms aimed to damage the protective cellular barriers and/or to modulate immune responses of the host to achieve invasion.

**Figure 4:**
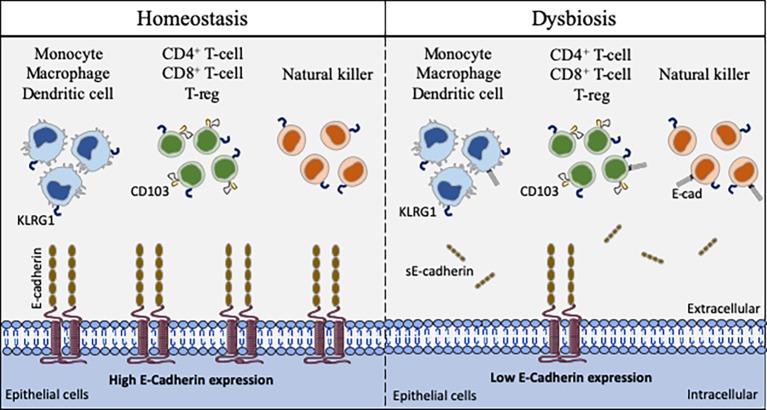
Schematic model of interaction between cells that express CD103, KLRG1, and E-cad. E-cad expressed on epithelial cells (as well as dendritic cells, Langerhans cells, and macrophages) can potentially interact with CD103, KLRG1 or both molecules expressed on the surface of immune cells. In the absence of bacterial infection, the epithelial cells express E-cad at high level, allowing immune cells to ensure the immune surveillance of epithelia (left panel). After pathogenic bacteria invasion, the cell-surface expression of E-cad is weak on epithelial cells due to the activation of sheddases and sE-cad is released in the epithelium microenvironment. The abnormal expression of E-cad on subpopulations of immune cells, the release of sE-cad, the weak expression of E-cad on epithelial cells likely contribute to immune system dysfunction at the bacterial invasion site (right panel).

We can question the place of the E-cad in this process of mobilization of immunity cells *via* trans homotypic (e.g., E-cad) or trans heterotypic (e.g., CD103 and KLRG1) interactions under normal and pathological conditions (Figure 4). Although E-cad is expressed by immature CD4^+^CD8^+^ thymocytes (Munro et al., 1996; Müller et al., 1997), after the thymocytes have left the thymus, E-cad is generally absent from most mature lymphocytes (Lee et al., 1994). Yet, under certain pathological conditions, cell-surface expression of E-cad was reported on mature T-lymphocytes (CD3^+^) subsets, as well as B cells (CD19^+^), NK cells (DX5^+^), and monocyte/macrophages (CD11b^+^) subpopulations (Esch et al., 2000; Sakai et al., 2008). E-cad expression was also confirmed for subpopulations of epithelial γδ T-cells (Lee et al., 1994) and memory CD8^+^ T-cells in intestinal mucosa (Hofmann and Pircher, 2011). Moreover, we have recently observed that 30% of CD16^+^ monocytes expressed E-cad after *C. burnetii* infection (Mezouar et al., 2019). In the intestinal epithelium, it is generally accepted that the immune host defense is mainly mediated by effector cells that express the α_E_ integrin (CD103), an E-cad ligand (Banh and Brossay, 2009; Van den Bossche et al., 2012). CD103 is expressed on 40–50% of CD4^+^ T-lymphocytes and 90% of CD8^+^ T-lymphocytes that reside in the intestinal mucosa as well as on the surface of intraepithelial lymphocytes (Cerf-Bensussan et al., 1987; Kilshaw and Murant, 1990; Hadley et al., 1997; Uchida et al., 2011). It is also present on T cells of the intestinal lamina propria (Agace et al., 2000) and a subset of CD4^+^CD25^+^ Foxp3^+^ Treg-cells (Stephens et al., 2007). It was demonstrated that after epithelial damages, the intraepithelial CD103^+^ γδ T-lymphocytes that reside on the surface of epithelium promote mucosal repair through antibacterial factors (e.g., Reg3γ) and immunomodulatory molecules (e.g., IL1β, CXCL9) (Cepek et al., 1994; Ismail et al., 2009, 2011). It is worth noting that the intestinal CD103^+^ intraepithelial lymphocytes adherence to epithelial cells is inhibited by antibodies against CD103 (Cepek et al., 1993). Another cell-surface receptor named KLRG1 that is encountered on subsets of immune cells including mature NK cells, memory CD4^+^T cells, effector CD8^+^ T cells, and FoxP3^+^ Treg cells, is known to bind E-cad (Schwartzkopff et al., 2007; Tessmer et al., 2007; Banh and Brossay, 2009; Van den Bossche et al., 2012). It has been reported that high levels of sE-cad could be sufficient to inhibit CD8^+^ T-cell function in a KLRG1-dependent manner (Streeck et al., 2011).

The E-cad induction on subpopulations of immune response cells in pathological situations remain to be elucidated. It can be hypothesized that the aberrant expression of E-cad under pathological conditions reflects changes in the transmigration and homing capacity of these cells (Reyat et al., 2017). Because several pathogenic bacteria reduce the epithelium surface expression of E-cad at the site of infection, it might be speculated that the decreased expression or the lack of expression of E-cad on epithelial cells is likely to trigger the rerouting of immune cells far from the infection site. As previously shown by Streeck et al. (2011) for the KLRG1^+^ CD8^+^ T-cells subpopulation, the release of sE-cad might also serve as a decoy for diverting from their function the immune cells expressing E-cad, CD103 or KLRG1 after engagement of such receptors with sE-cad. Modulation of E-cad expression on the host epithelial cells and sE-cad release could therefore be considered a very efficient stratagem to prevent the immune system from behaving as a line of defense against invaders. In addition, for bacteria that induce cancer, reducing the expression of E-cad on certain tumor cells as previously reported (Shields et al., 2019) and disrupting the migration and attachment capabilities of immune survey cells could be a way of promoting the development of bacteria-induced cancers.

## Conclusion and Discussion

**Figure 5:**
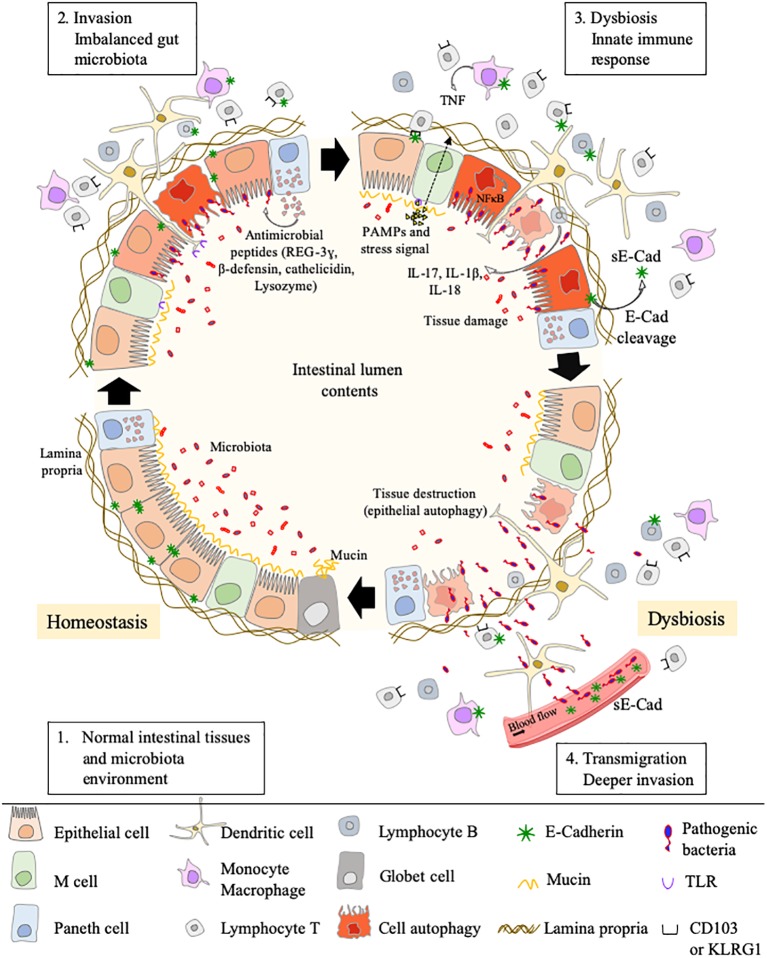
This cartoon illustrates how, by simply acting on the E-cad expression, an intestinal pathogenic bacterium can both bypass the physical defense system represented by the epithelial barrier and confuse the cells of the immune system intended to defend the host. Step 1: under homeostasis, the epithelium (composed of epithelial cells) is protected by a mucus layer synthesized by goblet cells which secrete the mucins (e.g, MUC2 – mucin gel). The mucus layer prevents microbial attachment without interference with the transport of nutrients. The commensal intestinal microbiota is limited to the epithelium-distal mucus layer, while the epithelium-proximal mucus is largely devoid of bacteria. Step 2: upon infection, the commensal intestinal microbiota competes with the infectious pathogens and the Paneth cells produce antimicrobial proteins (e.g., C-type lectin REG3γ, β-defensins, cathelicidins, and lysozyme), to fight the invasion. A regulation of infection is also achieved by epithelial cell autophagic clearance and dead cells renewal. At the same time, the villous microfold cells (M cells) expressing TLR deliver luminal antigens to the underlying immune system to set up a whole arsenal of anti-bacterial actions. In case this response proves sufficient, the invader is destroyed, and the microenvironment returns to homeostasis (Step 1). If not, the conflict is prolonged. Step 3: an efficient immune response against the pathogens requires the migration of cells of the host immune system in the microenvironment where the infection occurs and the sequential detection of stress signals, tissue damages, and PAMPs (e.g., lipopolysaccharide, flagellin, peptidoglycan, lipoproteins, and unique bacterial nucleic acid structures). KLRG1^+^ dendritic cells and monocytes/macrophages, CD103^+^ T-cells, KLRG1^+^ T-cells, and other immune cell subpopulations, colonize the lamina propria. Upon detection of bacterial invasion, host cell receptors, such as TLR and C-type lectin receptors, activate signaling pathways that govern the production of inflammatory cytokines, including the IL-1β and IL-18 that can restrict bacterial replication. Step 4: the pathogenic bacteria reduce the epithelium surface expression of E-cad at the site of infection, resulting in the destruction of adherent’s junctions and allowing transmigration. Moreover, it might be speculated that the induction of E-cad on subpopulation of immune response cells (E-cad^+^ T-cells and CD16^+^/E-cad^+^ monocytes) redirects those cells far from the infection site in microenvironments where they have a higher probability to interact with E-cad^+^ epithelial cells. The release of sE-cad might also serve as a decoy for diverting immune cells from their function through interaction with E-cad, CD103, or KLRG1 at the surface of immune cells.

This review highlights how the E-cad can be diverted from its function of maintenance of tissues integrity and prevention of cell migration/differentiation during pathogenic bacterial infections. Pathogenic bacteria can use E-cad for their attachment to epithelial cells. Indeed, they can cleave E-cad to ensure their transmigration and can modulate the responsiveness of immune cells through modulation of cell-surface expression of E-cad and sE-cad release in body fluids. Some bacteria use E-cad to enter their target cells (e.g., *F. nucleatum*, *L. monocytogenes*, *S. pneumoniae*). Several bacteria act on the cell-surface expression of this molecule, either by modulating the *CDH1* gene transcription (e.g., *C. trachomatis*, *H. pylori*) or by inducing the cleavage of the E-cad molecule (e.g., *C. perfringens*, *S. aureus*, *C. burnetii*) *via* proteases (sheddases). This process can favor achievement of the trans-epithelial host invasion or modulate host-pathogen molecular crosstalk. Currently, the best studied models are those that refer to intestinal infections that can lead to cancer (Figure 5).

Proteases (cysteine proteases, serine proteases, aspartate proteases, and metalloproteases) are ubiquitously encountered in the microbial world and are essential for their survival and replication cycle (Häse and Finkelstein, 1993). Proteases, such as collagenase (Bond and Van Wart, 1984), elastase (Bever and Iglewski, 1988), or metalloprotease (Schiavo et al., 1992) (Domann et al., 1992) were associated with bacterial pathogenesis. Some pathogenic bacteria can activate the production of eukaryotic proteases such as ADAM-10 *via* signaling (e.g., this was reported for *P. aeruginosa*, *S. marcescens*, *C. perfringens*, *S. aureus*), whereas others use a portion of their genome to encode their own sheddase, including the HtrA protease (encoded by *H. pylori*, *C. jejuni*, *S. flexneri*, enteropathogenic *E. coli*) or *B. fragilis* toxin (encoded by *B. fragilis*).

**Figure 6:**
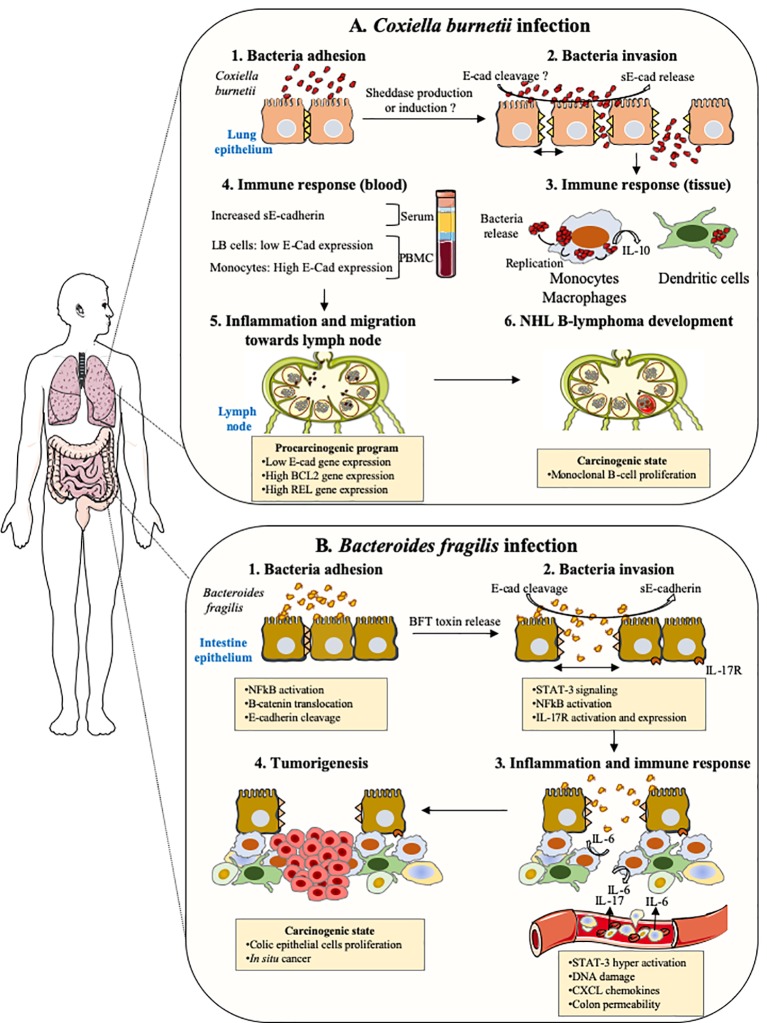
Cellular host E-cad as target during both pulmonary and intestinal bacterial infections. This drawing summarizes the hypothetical models of tumorigenesis associated with *Coxiella burnetii* and enterotoxigenic *Bacteroides fragilis*. **(A)** *Coxiella burnetii*. Although rare, the incidence of NHL B-lymphoma in patients infected by *C. burnetii* in France was significantly higher (25-fold) than within the general population. In Q fever, the overproduction of IL-10 by infected monocytes was found critical in both sustaining replication of the *C. burnetii* and preventing the macrophages microbicidal activity. Moreover, specific genes involved in anti-apoptotic process are over-expressed, whereas pro-apoptotic genes are repressed. Recently, we found elevated concentrations of sE-cad in Q fever patients, along with an increase in cell-surface expression of E-cad in more than 30% of HLADR^+^/CD16^+^ monocytes and a decrease in E-cad expression that concerned about 3% of the E-cad^+^/CD20^+^ B-cells subpopulation (LB cells). We speculate that the release of sE-cad might participate to the molecular crosstalk, which takes place in the microenvironment of the lymph node during persistent Q fever and might possibly trigger a pro-carcinogenic program required for the initiation of NHL lymphoma. **(B)** Enterotoxigenic *Bacteroides fragilis*. The enterotoxigenic *Bacteroides fragilis* synthesizes a toxin, BFToxin, which damages the protective intestinal epithelial barriers of the host by cleavage of E-cad to achieve invasion. This process leads to epithelial cells activation through nuclear translocation of β-cat and NF-κB, inducing the transcription of genes such as IL-17 receptor (IL17R). A pro-inflammatory immune response is initiated against the pathogen characterized by the *in-situ* production of IL-17. IL-17R positive cells are induced to produce STAT3 and a gradient of chemokines in the microenvironment favors the recruitment of pro-tumoral myeloid cells that accumulate in the distal colon producing growth factors triggering the proliferation of colic epithelial cells. These cells progressively accumulate DNA damages and form a solid tumor in the colon.

As described above in this paper, evidence emphasizing that cleavage of E-cad by sheddases and release of sE-cad into the body fluids are factors that contribute to the progression of cancer (sometimes it was demonstrated). Within the group of bacteria that modulate the expression of E-cad, the association with carcinogenic processes has been investigated, in particular with *H. pylori*, *F. nucleatum*, *S. gallolyticus*, and *B. fragilis*. *H. pylori* is known to be a risk factor for the development of gastric adenocarcinoma and the progression toward cancer is likely related to E-cad cleavage and β-cat activation (Murata-Kamiya et al., 2007), *F. nucleatum* promotes colorectal cancer by modulating the E-cad and Wnt/β-cat signaling *via* its FadA adhesin and up-regulating annexin A1 (Rubinstein et al., 2013, 2019). *S. gallolyticus* is also associated with colorectal cancer with known increased level of β-cat and c-Myc activation (Kumar et al., 2017). *B. fragilis* toxin (BFT)-mediated cleavage of E-cad initiates a multi-step inflammatory cascade requiring β-cat nuclear translocation, activation of NF-κB and Stat3 signaling pathways in colonic epithelial cells as early events leading to pro-tumoral myeloid cell infiltration to the distal colon and colon cancer (Chung et al., 2018). Our recent data indicate that *C. burnetii*, reported as associated with occurrence of non-Hodgkin lymphoma, is also capable of triggering cleavage of E-cad and release of sE-cad in the sera of Q fever patients (Figure 6; Mezouar et al., 2019). Yet, further experiments are required to formerly demonstrate the association between sE-cad release in sera from Q fever patients and the initiation of a pro-carcinogenic inflammatory process leading to lymphoma development.

The accumulation of data showing that sE-cad is produced in many pathological processes that can lead to cancer development raises the question of what value can be attributed to this compound as a biomarker of disease severity. At the moment, we do not have enough information to conclude. The presence of sE-cad in body fluids was considered a possible biomarker in *H. pylori* (O’Connor et al., 2011) and *C. burnetii* (Mezouar et al., 2019) infections, and soluble VE-cad was also regarded as possible biomarker in *E. coli* infections (Doulgere et al., 2015). We are currently implementing a test protocol to assess whether sE-cad could be a biomarker for tuberculosis and severe *C. difficile* infections. CD68^+^ granuloma macrophages from *M. tuberculosis* patients were reported to express E-cad (Cronan et al., 2016). Moreover, using *M. marinum* as model, Cronan and collaborators reported that the mycobacterial granuloma formation is accompanied by macrophage reprogramming that parallels E-cad-dependent mesenchymal-epithelial transitions and alters immune response. E-cadherin induction was found in the granuloma of both *M. marinum*-infected and uninfected granuloma macrophages, while macrophages residing outside the granuloma remained negative for E-cad. Concerning *C. difficile*, the etiologic agent of pseudomembranous colitis and severe diarrhea, it was reported that *C. difficile* TcdB toxin induces the redistribution of occludin and ZO-1without influencing the subjacent E-cad (Nusrat et al., 2001).

For the management of *H. pylori* gastrointestinal disorders and *H. pylori-associated* gastric cancer, it is recommended to use a combination of ranitidine bismuth citrate, clarithromycin, and amoxicillin (Goderska et al., 2018). In a near future, it will not be surprising to consider the possibility of proposing therapeutic approaches that will combine antibiotics, probiotics, and sheddase inhibitors to regulate E-cad as well as β-cat inhibitors. Probiotics (e.g., the yeast *Saccharomyces boulardii* CNCMI-745) have been used to restore intestinal barrier integrity in patients with inflammatory bowel disease, by regulation of E-cad recycling (Terciolo et al., 2017). Intensive research is aimed at developing inhibitors of MMPs (Antczak et al., 2008; Tuccinardi et al., 2008; Schmidt et al., 2016; Katoh, 2017) and ADAMs (Madoux et al., 2016). MMP-9 inhibitors that abrogate E-cad cleavage are considered a promising tool for therapeutic of colorectal cancers (Biswas et al., 2010; Marshall et al., 2015). This opens up new avenues of research for therapeutic purposes.

## Author Contributions

CD, SM and J-LM conceived the paper. SM and CD designed the tables and figures. CD wrote the paper.

### Conflict of Interest

The authors declare that the research was conducted in the absence of any commercial or financial relationships that could be construed as a potential conflict of interest.
